# Knowledge, attitudes and practices towards rabies: questionnaire survey in rural household heads of Gondar Zuria District, Ethiopia

**DOI:** 10.1186/s13104-015-1357-8

**Published:** 2015-09-02

**Authors:** Reta T. Digafe, Legesse G. Kifelew, Abraham F. Mechesso

**Affiliations:** Addis Ababa University, College of Veterinary Medicine and Agriculture, Bishoftu, Ethiopia; Department of Microbiology, St Paul’s Hospital Millennium Medical College, Addis Ababa, Ethiopia; School of Veterinary Medicine, Hawassa University, Hawassa, Ethiopia

**Keywords:** Knowledge, Practices, Questionnaire, Rabies, North Gondar

## Abstract

**Background:**

Rabies is a fatal animal disease of significant public health importance. Domestic dogs are the main reservoir and transmitter of this disease particularly in developing countries. Even though rabies is a highly fatal disease, it is a preventable disease. Community awareness about rabies is one of the key components for prevention. This study describes the knowledge, attitudes and practices of a rural community in Gondar Zuria District, Ethiopia.

**Methods:**

A cross sectional study was conducted from March to June, 2013. A structured questionnaire was used to collect the data through face to face interviews among 400 respondents. The data were then analyzed using SPSS statistical software version 20.

**Results:**

The current study indicated that almost all (99.3 %) of the surveyed individuals were aware of the disease rabies. Rabies is considered to be a fatal disease in humans by 67.8 % of the respondents while 27.8 % believe that it is a treatable disease. Dogs were indicated as source of infection for humans by all respondents followed by equines (27.2 %) and cats (12.1 %). Bite was known as mode of rabies transmission by majority of the respondents (94 %) while other means were given less weight. Aggression was described as a major clinical sign of rabies in animals. Consumption of cooked or boiled meat from rabid animals was considered as safe by 67.0 % of the respondents and about 19 % replied even raw meat is safe for human consumption. The need for immediate treatment after exposure was mentioned by less than half (47.4 %) of the respondents and only 38.8 % of the respondents considered modern medicine as appropriate treatment after exposure to rabid animals. Nearly 42 % of respondents had experienced a dog bite. Following the dog bites, only 30.7 % practiced washing of the wounds with water as first aid.

**Conclusion:**

Rabies was found to be well known in the study area. However, knowledge and practices in prevention of rabies were limited. Education of rabies about possible sources of infection, mode of transmission and measures to be taken after exposure is very important in the study area.

**Electronic supplementary material:**

The online version of this article (doi:10.1186/s13104-015-1357-8) contains supplementary material, which is available to authorized users.

## Background

Rabies is one of the most serious zoonotic diseases. It is almost 100 % fatal once the clinical signs develop [1, 2]. The causative agent belongs to the genus *Lyssavirus* of the family *Rhabdoviridae* [3]. Rabies is the most widely recognized example of salivary transmission of viruses. Inoculation of infected saliva through the bite of a rabid animal appears to be the predominant mode of rabies viral entry although contamination of broken skin and mucous membrane such as mouth, nasal cavity or eyes by fresh saliva or neurological tissues may result in infection [4].

Africa is, after Asia, the second continent most affected by rabies with an estimated 24,000 (44 %) of the 55,000 annual rabies deaths worldwide [5]. The burden of rabies falls mostly on poor rural communities and children in particular [6]. In Ethiopia, rabies is an endemic disease with a high incidence rate [7–9]. It has been diagnosed from various species of domestic and wild animals. However, available evidences suggest that domestic dogs are the main reservoir and responsible species for human cases in the country [10–12].

Deaths from rabies could be prevented by the timely application of appropriate prophylaxis [13]. Community awareness about rabies is very crucial in rabies prevention and control [6]. For efficiently increasing awareness, the knowledge gap among the community should be identified and targeted. We thus conducted a questionnaire survey to assess the level of knowledge, attitudes, and practices regarding rabies in rural household heads of Gondar Zuria District, Northern Ethiopia.

## Methods

### Study site

**Fig. 1:**
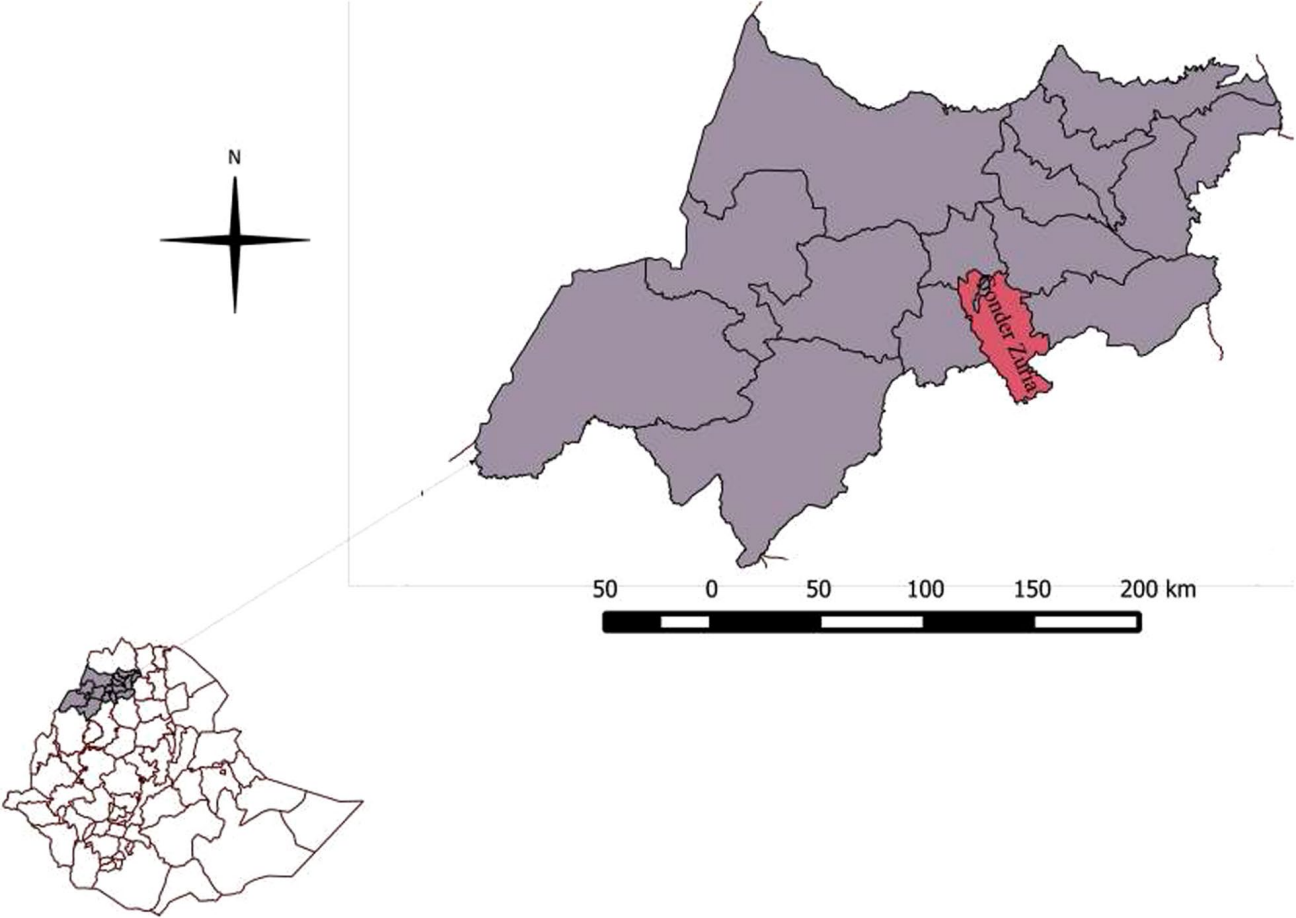
Map of Gondar Zuria District

This study was conducted from March to June, 2013 in Gondar Zuria District, North West Ethiopia. The district has a total human population of 222, 377 (112,248 were males and 110,129 were females) [14]. Ninety percent of the population in the district are rural inhabitants [15]. The district comprises 35 ‘Kebeles’ (the smallest administrative unit) and the study was conducted in 13 of them. Map of Gondar Zuria district is shown in Fig. 1.

### Study design

A cross sectional study design was used in this study. Thirteen animal health workers who were diploma and bachelor degree holders in animal health disciplines were recruited to collect the data. Before beginning data collection, adequate training was given to the data collectors on rabies and data collection procedures. The questionnaire, originally prepared in English was translated to the local language, Amaharic. This questionnaire was administered to 10 randomly selected individuals in the study area to check the understandability comprehension of the questions, and simultaneously it was used as part of the data collection training for data collectors. Ambiguous words were made clearer based on the feedback. Individuals involved in the pretest were not included as the study participant. The data were collected through face-to-face interviews. The data were then analyzed using SPSS statistical software version 20. The results are described in percentages.

### Sample size determination and sampling techniques

Sample size was determined using Cochran’s sample size formula for categorical data [16].

$${\text{n }} = \frac{{\left( {\text{t}} \right)^{ 2} *{\text{P }}\left( {\text{q}} \right)}}{{{\text{d}}^{ 2} }}\;\;\;\;\;\;\;\;{\text{n }} = \frac{{\left( { 1. 9 6} \right)^{ 2} 0. 5 { }\left( { 1- 0. 5} \right)}}{{\left( {0.0 5} \right)^{ 2} }} = 3 8 4 { }$$

where t is the value for selected alpha level of 0.025 in each tail = 1.96. (p), (q) is the estimate of variance = 0.25, d is the acceptable margin of error = 0.05.

By adding 10 % non response rate, the final sample size estimated was 422.

Out of 35 ‘Kebeles’ in the district, 13 of them were included in this study. These sites had veterinary professionals assigned by the authority of the district in attempt to have better veterinary service coverage. The veterinary professionals were targeted for data collection to get quality data efficiently with less cost, ease of communication for the supervisors and high response rate. In parallel, the professionals were updated about rabies so that they were ready to clarify any ambiguities in the questionnaire and answer other rabies related questions that may be raised during the interviews.

Almost equal numbers of household heads were selected from each Kebele. Briefly, 30 and 33 individuals per kebele were selected from 10 and 2 kebeles respectively. The remaining 34 individuals were selected from 1 Kebele. The total population in the district is estimated to be 222, 000 with an average of six members per household. Systematic random sampling was used in selecting the houses by choosing every 30th house with an estimated 1000 households per Kebele. From each household, a person who economically supports or manages the household (heads of the family) was interviewed. The limitation with this sampling approach is the fact that remote inhabitants from areas not covered by the veterinary clinics in the district may be missing or under-represented. This could be improved through increasing the study area coverage and number of study participants.

### Ethical clearance

The study protocol was reviewed and approved by Institutional Review Board of University of Gondar Research and Community Service Office. Oral informed consents were obtained from each participant after informing them about the purpose of the study as well as the risks, benefit and rights of the study participants. Only voluntary participants were involved in the study. All the information obtained from the study participants was kept confidential.

## Results

A total of 400 heads of household were interviewed in this research. The ages of the respondents range from 20 to 86 with a mean age of 44.47 ± 12.33. More than ninety percent (90.3 %, n = 361) of them were males. About 40 % (n = 159) of the respondents were illiterate, 26.5 % (n = 106) do write and read, 29.7 % (n = 119) had primary to secondary education and very few (4 %, n = 16) had a higher education.

Ninety-nine point three percent (n = 397) of the respondents had heard of rabies and similarly, a high proportion (87 %, n = 348) of them reported that they had encountered rabid animal(s) in their life time at least once. From those individuals who had seen rabid animals, 90.8 % (n = 316) replied that the animals had died or been killed; 6.9 % (n = 24) said the animals had recovered. From those 24 respondents that reported the rabid animals had recovered, a majority (58.3 %, n = 14) of them said the animals recovered after they had been given “Holy water” (a spiritual practice). The remaining respondents mentioned animals recovered following traditional medicine, modern medicine, traditional and spiritual practice, and modern and spiritual practice. Furthermore, 53.5 % (n = 214) of respondents reported that they had seen rabid humans. In this paper, Holy water (a spiritual practice) refers to a water that a priest has blessed, locally termed as “Tsebel”. Traditional medicine refers to herbal medicine given by known traditional healers and it is taken orally while modern medicine refers to wound management and post exposure vaccination in health care facilities by medical professionals.

Generally, rabies was considered to be a veterinary and public health problem in the study area by 92.3 % (n = 369) of the respondents. The case fatality rate in humans was reported to be 66.8 % (n = 143). A considerable proportion, 33.2 % (n = 71), replied that the patients had recovered from the illness. Respondents mentioned that patients recovered following traditional medicine, spiritual practice, modern medicine or the combination of any two of them. Despite their observation, when asked about their perception of the fatality rate of rabies, 67.8 % (n = 271) believe that rabies is fatal after clinical signs developed in humans. More than a quarter (27.8 %, n = 111) of the respondents believe that it is a treatable disease. Details of the knowledge and perceptions summarized above are described in Additional file 1: Table S1.

**Fig. 2:**
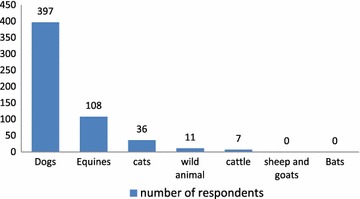
Source animals for rabies

Dogs were mentioned as the source of infection for humans by all respondents that had heard of rabies. Very few (1.8 %, n = 7) individuals replied cattle and none of them mentioned sheep, goats and bats as sources of infection (see Fig. 2).

**Fig. 3:**
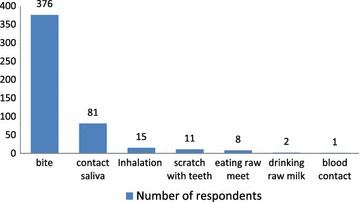
Modes of rabies virus transmission

Bite was reported by 94 % (n = 376) of the surveyed people as the mode of transmission for rabies followed by contact with saliva. Scratch, eating raw meat and drinking raw milk were also mentioned by limited respondents. Inhalation was also indicated as means of transmission for rabies as depicted in Fig. 3.

**Fig. 4:**
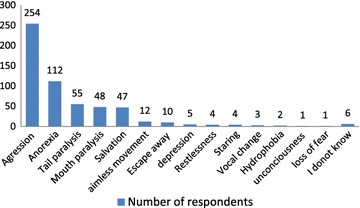
Clinical signs of rabies in animals

The most frequently mentioned incubation period by the respondents for both animals and humans was 40 days while the range was 3–90 days for animals and 3–180 days for humans. Aggression was pointed out as a major clinical sign in rabid animals by the majority (63.5 %, n = 254) of the respondents followed by anorexia (28.0 %, n = 112), downward dropping of tail (13.8 %, n = 55), open mouth (12.0 %, n = 48) and salvation (11.8 %, n = 47) as shown in Fig. 4.

When asked about disposal of food animals affected with rabies, the majority 67.0 % (n = 268) of the respondents believe cooked or boiled meat is safe for human consumption and about one-fifth mentioned even consumption of raw meat is safe. Less than a quarter (23.3 %, n = 93) replied burying or burning the carcass of animals died of rabies is appropriate way of disposal as summarized in Additional file 2: Table S2.

Fewer (44.8 %, n = 179) of the respondents mentioned the need for immediate treatment after exposure to rabies-suspected animals. More than a quarter (28.5 %, n = 114) of the respondents replied it is safe to look for treatment within 2–7 days. Up to 40 days were considered not too late to look for treatment in this study. When asked about treatment options after exposure to rabies, only 38.8 % (n = 155) of the respondents consider modern medicine to be appropriate treatment after exposure to rabid or rabies suspected animals. About one-third (35 %, n = 140) prefer traditional medicine. In addition, Holy water, traditional medicine with holy water; traditional medicine with modern medicine; and holy water with modern medicine were mentioned as a preference of treatment by the respondents. Time before treatment is sought and treatment preference is summarized in Additional file 3: Table S3.

Just over forty-one percent (41.5 %, n = 166) of respondents had experienced a dog bite at least once in their life. Following a dog bite less than one-third (30.7 %) had practiced washing of the wound with water or soap and water as first aid to prevent rabies. Others had applied traditional medicine, did nothing as a treatment, or went to health facilities. Surprisingly, 86.8 % (n = 347) had not received any form of education by professionals about what to do if bitten by rabid or rabies suspected animals. The numbers are summarized in Additional file 4: Table S4.

## Discussion

The current study revealed that almost all respondents were aware of rabies. A High proportion (87 %) of them reported that they had encountered a rabid animal in their life time at least once. In line with our report, another study indicated that 98.6 % of people from a rural community of Gujarat, India [17] were aware about rabies was reported. Jemberu et al. [7] also reported a high (98 %) level of awareness about rabies in Gondar Zone, Ethiopia. However, the current investigation is higher than other reports from Addis Ababa, Ethiopia and India that reported 83 and 68.7 %, respectively [18, 19]. The reason for the discrepancy could be due to real difference in incidence of rabies in the study areas. In our study, the study participants were heads of household living in rural areas. This group has better communication and information about what is happening in their residential area, including animal disease situations, which may contribute to their high level of awareness.

The case fatality rate for rabies is almost 100 % once clinical signs are established both in domestic animals and humans [1, 20]. A lower level of case fatality rate was reported by the respondents in this study. In comparison, the respondents indicated that there was a higher (90.8 %) fatality rate in rabid animals than humans (66.8 %). The lower case fatality rate reported by the respondents compared to what is shown by the preponderance of scientific evidence might arise from misclassification of other diseases as rabies. Additionally, the higher fatality reported in animals suggests that the study participants have better knowledge in identifying rabies in animals than in humans. In this study, 10 % recovery in animals and 33.2 % in humans were believed to be mainly attributed to spiritual practices and/or traditional medicine although to date there are no known drugs for rabies treatment. The belief that rabies is treatable will most likely be a challenge in education campaigns. It is critical to convince the community that the disease is totally fatal and non-treatable once the clinical signs are manifested in order to encourage to look for treatment before the occurrence of the symptoms. Generally, rabies was considered as a veterinary and public health problem in the study area by more than 90 % of the respondents. This is reasonable for the study area where almost all people have heard of rabies and this provides a good opportunity to implement rabies prevention, control and elimination programs.

Dogs were mentioned as the source of infection for humans by almost all respondents. Equines were mentioned most after dogs. Very few individuals mentioned cattle as a source for transmission of rabies to humans and none of them mentioned sheep, goats and bats in the current study. In fact, dogs are known to be responsible for more than 90 % of all human rabies cases worldwide [21]. Horses and donkeys have been shown to be aggressive and bite ferociously when they are rabid [13]. Cattle have not been shown to bite when they are rabid [22]. If this is true generally, this might have contributed to the perception of many participants to consider equines as the highest source of infection after dogs.

In this study, bite was mentioned as mode of transmission for rabies to humans by a large majority of the respondents. Considerable percent of participants mentioned contact with saliva as mode of transmission. Inhalation and scratch with teeth were also considered as means of transmission by a few of the respondents. Very few mentioned milk, meat consumption, and contact with blood as mode of transmission. Inoculation of infected saliva through the bite of a rabid animal appears to be the predominant mode of rabies transmission [2]. Contact of infected saliva with broken skin or mucous membrane can transmit the disease and also consumption or preparation of meat from rabid animals is a risk [13]. The virus is not carried in blood [6]. Therefore, transmission through blood contact is unlikely. It is under special conditions have infections occurred through aerosols, (e.g., in caves inhabited by bats and by exposure in the laboratory) [23, 24]. Even though the extent of transmission varies, all possible modes of transmission including bite, contact with saliva, and consumption of animal products from diseased animal should be avoided.

The most frequently mentioned incubation period by the respondents for both animals and humans was 40 days though the range was 3–180 days. Previous observations indicated that incubation period for rabies is highly variable ranging from as few as 10 days to several years influenced by various factors including host species, virus strain, amount of inoculum and the site of introduction of the virus [25]. The perception that the incubation period is long may hinder the exposed individual from taking prompt action to seek medical care. On the other hand, those who believe the incubation period is short may not seek post exposure prophylaxis once the perceived incubation period has passed. This is serious for rabies-infected individuals where mortality is 100 % after development of clinical signs.

Our study showed that there was good level of awareness regarding clinical signs of rabies. Aggression was mentioned as a clinical sign by majority of the respondents which is in line with the fact that furious form of rabies is the common type of rabies in animals [26–28]. This form of rabies is the more easily identifiable clinical form by most people and attracts individuals’ attention as the animals tend to attack humans and other animals. Rabies is known as mad dog in the community which is associated with the clinical sign, aggression. The paralytic form may be overlooked by the community since it may not be noticed by those who associate rabies only with madness i.e. aggression. Under such condition people may not seek post exposure prophylaxis when exposed to animals with the paralytic form of the disease and thus may risk death by rabies.

A low level (38.8 %) of preference for anti-rabies PEP was observed in this study. Most respondents choose other options like traditional medicine and/or spiritual practices. Similarly, studies conducted in Addis Ababa, Ethiopia, reported 58.3 % participants of the study participants had strong beliefs in traditional medicine [18]. Jemberu et al. reported even higher (84 %) reliance of respondents on traditional treatment. The preference for traditional practices might be arise from many factors including easy access to traditional medicine, lack of awareness, long duration of treatment and potential side effects of the Fermi type vaccine widely available and used as anti-rabies post exposure treatment in Ethiopia. Reliance on traditional medicines with unproven efficacy is very risky in that nothing can be done to save one’s life after the first symptoms of the disease occur. After suspected or proven exposure to rabies virus, immediate use of efficient anti-rabies vaccine with proper wound management and simultaneous administration of rabies immunoglobulin is almost invariably effective in preventing rabies [6].

Nearly 42 % of the respondents had experienced dog bites, but following the dog bite only fewer than one-third had practiced washing of the wounds with water or soap and water as first aid to prevent rabies. Other published surveys have indicated that similar proportion of people felt that washing the wound with soap and water was the best option [17, 19]. Washing of rabies-infected wounds with soap and water can increase survival by 50 % [2]. This treatment is cheap, readily available and feasible for all to apply.

Surprisingly, most of the participants had not received any form of education by professionals on what to do if bitten by rabid or rabies suspected animals. This strongly suggests that rabies is still a neglected disease, at least in the study area, and much has to be done by health and veterinary professionals so that prevention of rabies becomes a priority.

## Conclusions

Rabies is a well known disease in the study area and is considered to be a disease of significant public health importance. The level of knowledge about the main mode of transmission (animal bite) and source animal (dog) for rabies is good. However, contact with infected saliva and scratch with teeth should also be considered as a high risk. All mammals should be considered as a potential source of infection and care should be taken in handling these animals if appear sick. On the other hand, there is a lack of knowledge about what to do after exposure, like wound washing, immediate visits to health facilities, and use of anti-rabies post exposure prophylaxis. This might be mainly due to lack of education about the disease in the community. Therefore, continuous and strategic community awareness programs are very critical to prevent human cases in the current study area. Local traditional healers and spiritual leaders may play a great role in addressing education of the community as many individuals rely on their practices. Veterinary and health professionals should give due attention to increasing rabies awareness and prevention measures in the communities. A long-term strategy should target eradication of canine rabies.

## Additional files

Additional file 1:
**Table S1.** Knowledge and perceptions about rabies in Gondar Zuria District. Additional file 2:
**Table S2.** Perceptions about disposal of rabid food animals. Additional file 3:
**Table S3.** Time to look for treatment and treatment preferences. Additional file 4:
**Table S4.** Dog bite, response to dog bite and education about rabies.
