# Supplementary material for: Knowledge, attitudes and practices towards rabies: questionnaire survey in rural household heads of Gondar Zuria District, Ethiopia
Source: BMC Res Notes. 2015 Sep 2;8:400. doi: 10.1186/s13104-015-1357-8 (PMC4566865; doi:10.1186/s13104-015-1357-8)
Supplement: Additional file 1: — Table S1. Knowledge and perceptions about rabies in Gondar Zuria District. [file 13104_2015_1357_MOESM1_ESM.pdf]

Table 1: Knowledge and perception about rabies in Gondar Zuria district

Variables	Response	Number	Percent
Heard of rabies	Yes	397	99.3
	No	3	0.7
Saw rabid animal(s)	yes	348	87
	No	16	13
Observed rabid animal(s)	Died/killed	316	90.8
	Recovered	24	6.9
	Do not know	3	0.9
	Died & recovered	5	1.4
Treatment given for the recovered animal	Holy water	14	58.3
	traditional medicine	4	16.7
	modern medicine	3	12.5
	traditional and holy water	2	8.3
	modern and holy water	1	4.2
Saw rabid humans	Yes	214	53.5
	No	186	46.5
Observed rabid Human(s)	Died	143	66.8
	Recovered	71	33.2
Treatment given for the recovered person	traditional medicine	30	42.3
	holy water	11	15.5
	modern and holy water	8	11.3
	traditional and holy water	5	7
	modern and traditional medicine	4	5.6
	Do not know	13	18.3
Is rabies treatable	yes	111	27.8
	No	271	67.8
	I do not know	18	4.5
