# Supplementary material for: Knowledge, attitudes and practices towards rabies: questionnaire survey in rural household heads of Gondar Zuria District, Ethiopia
Source: BMC Res Notes. 2015 Sep 2;8:400. doi: 10.1186/s13104-015-1357-8 (PMC4566865; doi:10.1186/s13104-015-1357-8)
Supplement: Additional file 2: — Table S2. Perceptions about disposal of rabid food animals. [file 13104_2015_1357_MOESM2_ESM.pdf]

Table 2: Perception about disposal of rabid food animals

Variables	number	percent
Consumption of cooked or boiled meat is safe	268	67
Consumption of raw meat is safe	76	19
Burying or burning	93	23.3
Dispose dead animal in open field	19	4.8
Do not know	20	5
