# Supplementary material for: Knowledge, attitudes and practices towards rabies: questionnaire survey in rural household heads of Gondar Zuria District, Ethiopia
Source: BMC Res Notes. 2015 Sep 2;8:400. doi: 10.1186/s13104-015-1357-8 (PMC4566865; doi:10.1186/s13104-015-1357-8)
Supplement: Additional file 3: — Table S3. Time to look for treatment and treatment preferences. [file 13104_2015_1357_MOESM3_ESM.pdf]

Table 3: Time to look for treatment and treatment preference

Variable		Number	Percent
Time to look for treatment	Immediately	179	44.8
	2 to 7 days	114	28.5
	8 to 40 days	85	21.3
	Do not know	2	5.5
Preference of treatment	modern medicine	155	38.8
	traditional medicine	140	35
	Holy water	56	14
	traditional medicine with holy water	24	6
	traditional medicine with modern medicine	10	2.5
	Holy water with modern medicine	10	2.5
