# Supplementary material for: Knowledge, attitudes and practices towards rabies: questionnaire survey in rural household heads of Gondar Zuria District, Ethiopia
Source: BMC Res Notes. 2015 Sep 2;8:400. doi: 10.1186/s13104-015-1357-8 (PMC4566865; doi:10.1186/s13104-015-1357-8)
Supplement: Additional file 4: — Table S4. Dog bite, response to dog bite and education about rabies. [file 13104_2015_1357_MOESM4_ESM.pdf]

Table 4: Dog bite, response to dog bite and education about rabies

Variables	Response	Number	Percent
Dog bite	Yes	166	41.5
	No	234	57.5
Action taken after bite	washing of the wound with water/ soap and water	51	30.7
	Traditional medicine	53	31.9
	Did nothing	33	19.9
	Went to health facilities	20	12.1
Education about rabies	No	347	86.8
	Yes	53	12.6
